# Conformational comparisons of *Pasteurella multocida* types B and E and structurally related capsular polysaccharides

**DOI:** 10.1093/glycob/cwad049

**Published:** 2023-06-19

**Authors:** Nicole I Richardson, Neil Ravenscroft, Michelle M Kuttel

**Affiliations:** Department of Chemistry, University of Cape Town, Rondebosch 7701, South Africa; Department of Chemistry, University of Cape Town, Rondebosch 7701, South Africa; Department of Computer Science, University of Cape Town, Rondebosch 7701, South Africa

**Keywords:** antigen conformation, capsular polysaccharide, cross reactivity, molecular modeling, Pasteurella multocida

## Abstract

*Pasteurella multocida*, an encapsulated gram-negative bacterium, is a significant veterinary pathogen. The *P. multocida* is classified into 5 serogroups (A, B, D, E, and F) based on the bacterial capsular polysaccharide (CPS), which is important for virulence. Serogroups B and E are the primary causative agents of bovine hemorrhagic septicemia that is associated with significant yearly losses of livestock worldwide, primarily in low- and middle-income countries. The *P. multocida* disease is currently managed by whole-cell vaccination, albeit with limited efficacy. CPS is an attractive antigen target for an improved vaccine: CPS-based vaccines have proven highly effective against human bacterial diseases and could provide longer-term protection against *P. multocida*. The recently elucidated CPS repeat units of serogroups B and E both comprise a N-acetyl-β-D-mannosaminuronic acid/N-acetyl-β-D-glucosamine disaccharide backbone with β-D-fructofuranose (Fru*f*) side chain, but differ in their glycosidic linkages, and a glycine (Gly) side chain in serogroup B. Interestingly, the *Haemophilus influenzae* types e and d CPS have the same backbone residues. Here, comparative modeling of *P. multocida* serogroups B and E and *H. influenzae* types e and d CPS identifies a significant impact of small structural differences on both the chain conformation and the exposed potential antibody-binding epitopes (Ep). Further, Fru*f* and/or Gly side chains shield the immunogenic amino-sugar CPS backbone—a possible common strategy for immune evasion in both *P. multocida* and *H. influenzae*. As the lack of common epitopes suggests limited potential for cross-reactivity, a bivalent CPS-based vaccine may be necessary to provide adequate protection against *P. multocida* types B and E.

## Introduction

*Pasteurella multocida* is a widespread pathogenic encapsulated gram-negative bacterium that primarily infects animals (Harper et al. 2006; Wilkie et al. 2012). The *P. multocida* causes a range of diseases in different host species: from fowl cholera in poultry, through hemorrhagic septicemia in cattle and buffaloes, atrophic rhinitis in swine, to snuffles in rabbits. Further, exposure to animals (e.g. through licks, bites, and scratches) causes occasional severe zoonotic infections with *P. multocida* in humans (Wilson and Ho 2013). Bovine hemorrhagic septicemia disease due to *P. multocida* is of particular concern due to the significant economic losses incurred worldwide, especially in low- and middle- income countries in Asia and sub-Saharan Africa where veterinary care is limited (Benikrane and De Alwis 2002). As antibiotics are only effective if started in the very early stages of disease—a challenge in environments with limited resources (Benikrane and De Alwis 2002; Lubroth et al. 2007)—hemorrhagic septicemia is primarily managed through relatively inexpensive whole-cell killed veterinary vaccines. However, these provide limited short-term protection of 6–12 months (Lubroth et al. 2007; Wilson and Ho 2013; Capik et al. 2021); more effective vaccines against *P. multocida* could therefore have significant benefit.

The *P. multocida* is classified into 5 serogroups (A, B, D, E, and F) based on the capsular polysaccharide (CPS) and 16 serovars based on cell wall lipopolysaccharide (Heddleston et al. 1972; Carter 1984). The capsular serogroups largely determine the hosts and diseases, with serogroups B and E associated with bovine hemorrhagic septicemia, serogroups A and F associated with fowl cholera, and serogroups D and A associated with porcine atrophic rhinitis (Wilson and Ho 2013). The CPS is an attractive vaccine antigen target, as it is essential for virulence of *P. multocida* and inhibits phagocytic uptake (Boyce and Adler 2000; Aktories et al. 2012).

**Fig. 1:**
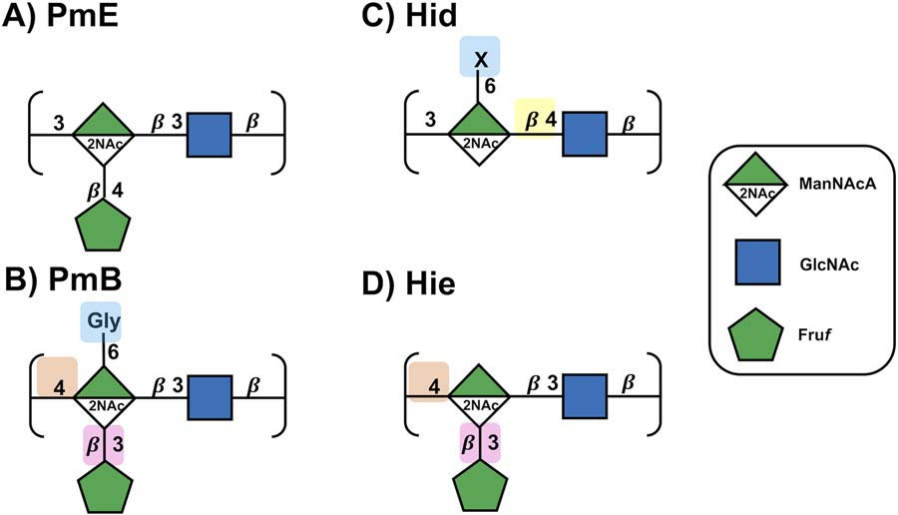
CPS structures for (A) PmE, (B) PmB, (C) Hid and (D) Hie represented with the SNFG symbol nomenclature for glycans (Varki et al. 2015; Haltiwanger 2016; Neelamegham et al. 2019). Differences in RUs relative to PmE are indicated by shading of the background.

The recent structural elucidation of the *P. multocida* serogroup B (PmB, Fig. 1A) and E (PmE, Fig. 1B) (St Michael et al. 2021) CPSs renews interest in the CPS as a target antigen for a bovine vaccine against *P. multocida*. The CPSs in these serogroups are structurally similar (Table 1). The repeat unit (RU) comprises N-acetyl-$\mathrm{\beta}$-D-mannosaminuronic acid (ManNAcA), N-acetyl-$\mathrm{\beta}$-D-glucosamine (GlcNAc), and $\mathrm{\beta}$-D-fructofuranose (Fru*f*): ManNAcA and GlcNAc form a disaccharide backbone, with a Fru*f* side chain linked to ManNAcA (St Michael et al. 2021). The serogroups differ in 3 respects (indicated by shaded boxes in Fig. 1): the GlcNAc $\mathrm{\beta}$(1→3) linkage in PmE is replaced with a $\mathrm{\beta}$(1→4) linkage in PmB; the Fru*f* side chain β(2→4) linkage in PmE is replaced with a β(2→3) linkage in PmB; and PmB contains an N-linked glycine (Gly) side chain at C6 of ManNAcA.

**Table 1:** Line structures of PmB and PmE as well as Hie and d (Hid) RU CPSs. (Side groups indicated in bold).

Organism	Antigen RU structure in CASPER format
PmE	{➔3)[$\boldsymbol{\mathrm{\beta}}$**DFru*f*(2➔4**)]$\mathrm{\beta}$DMan*p*NAcA(1➔3)$\mathrm{\beta}$DGlc*p*NAc(1➔}*_n_*
PmB	{➔4)[$\boldsymbol{\mathrm{\beta}}$**DFru*f*(2➔3)]**$\mathrm{\beta}$DMan*p*NAcA6**Gly**(1➔3)$\mathrm{\beta}$DGlc*p*NAc(1➔}*_n_*
Hie	{➔4)[$\boldsymbol{\mathrm{\beta}}$**DFru*f*(2➔3)**]$\mathrm{\beta}$DMan*p*NAcA (1➔3)$\mathrm{\beta}$DGlc*p*NAc(1➔}*_n_*
Hid	{➔3)$\mathrm{\beta}$DMan*p*NAcA6***X***^a^(1➔4)$\mathrm{\beta}$DGlc*p*NAc(1➔}*_n_*

^a^ *X* represents a variable amino acid moiety: either L-alanine, L-serine, or L-threonine in the proportions 2:2:1.

Interestingly, PmE and PmB are structurally similar to the *Haemophilus influenzae* type e (Hie, Fig. 1C) and d (Hid, Fig. 1D) CPS, a possible example of convergent evolution. The *H. influenzae* is a leading cause of meningitis, otitis media among other infections in children (Richardson, Kuttel, et al. 2021). Type b was the primary cause of disease prior to the development of a vaccine; however, we are now seeing an increase in nonvaccine disease, especially due to type a with Hie and *H. influenzae* type f also showing increases (Slack 2021). Hie is very similar to PmB, lacking only the Gly substituent (Branefors-Helander et al. 1981a; Tsui et al. 1981), while Hid has a different backbone glycosidic linkage pattern (3-linked ManNAcA and 4-linked GlcNAc), lacks a Fru*f* substituent, and has a variable N-linked amino acid substitution pattern on C6 of ManNAcA (L-alanine, L-serine, L-threonine in the proportion 2:2:1) (Branefors-Helander et al. 1981b; Tsui et al. 1981).

An understanding of the potential for cross-reactivity (and hence cross-protection) between related target antigens—which is believed to have both structural and conformational aspects—may assist in the design of a minimal valency vaccine that provides maximum disease coverage (Kuttel and Ravenscroft 2018). The structural similarity between PmE and PmB points to the potential for cross-reactivity between these antigens, which would allow for a cheaper monovalent bovine vaccine. Early work using purified CPS in cattle challenge and passive mouse protection studies supports low levels of heterologous cross-reactivity and protection with good homologous serotype protection (Penn and Nagy 1974, 1976; Nagy and Penn 1976). A more recent study, in which the CPS structures were elucidated for the first time, showed cross-reactivity between a PmB conjugate vaccine and PmE CPS, with limited cross-protection against PmE bacteria in mice (St Michael et al. 2021). The reciprocal study, however, was not performed and further work is still underway to extend these data (St Michael et al. 2021). In the absence of more detailed experimental information on cross-reactivity, molecular modeling can provide potentially useful information on which epitopes are exposed and are therefore likely to be recognized by antibodies (Richardson, Kuttel, et al. 2021; Richardson et al. 2022). While not a current vaccine target, modeling the structurally related Hie and Hid CPS provides insight into the conformational effect of structural differences.

**Fig. 2:**
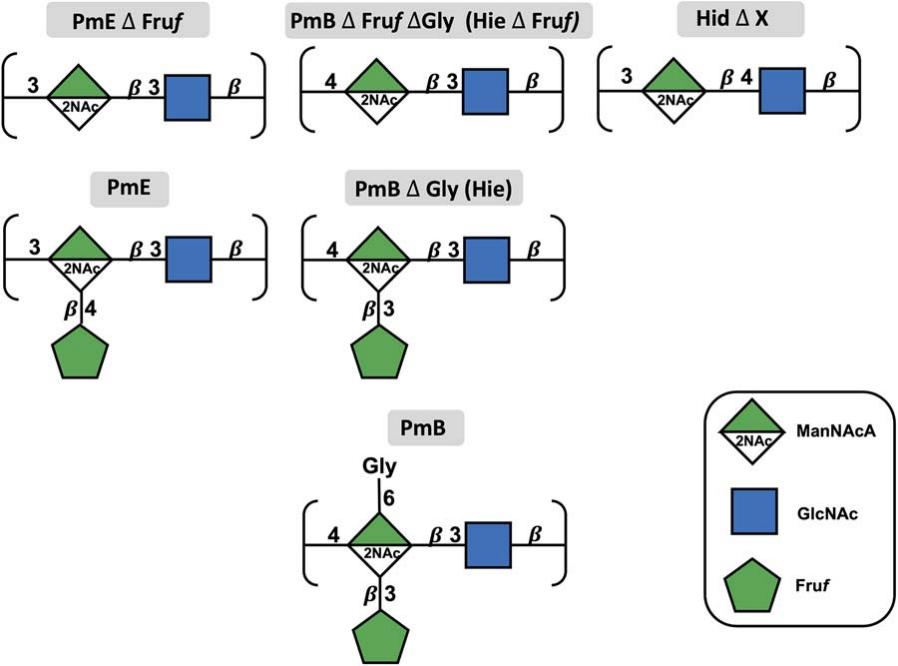
CPS molecules of PmE and B (PmB) and Hid and Hie modeled in this work (SNFG symbol representation). The top panel shows the unsubstituted backbone molecules: PmE without Fru*f* (PmE $\Delta$ Fru*f*), PmB without Fru*f* and Gly (PmB $\Delta$ Fru*f*  $\Delta$ Gly equivalent to Hie without Fru*f*), and Hid without the variable amino acid substituent (L-alanine, L-serine, or L-threonine in the proportions 2:2:1) represented by *X* (Hid $\Delta$  *X*). The middle panel shows the modeled Fru*f*-substituted molecules: PmE wild type, PmB without Gly (PmB $\Delta$ Gly; equivalent to Hie wild type). The bottom panel shows the wild-type PmB, with the Gly substituent (PmB).

Here, we report a comparative modeling study to assess the conformational impact of the different structural features in the similar PmE, PmB, Hie, and Hid CPS antigens. We first compare the unsubstituted CPS backbones (PmE $\Delta$ Fru*f*, PmB $\Delta$ Fru*f*  $\Delta$ Gly, and Hid $\Delta$  *X*—where *X* is the variable amino acid described above) to determine the effect of changes in the glycosidic linkages on the chain conformation (Fig. 2, top panel). Next, we investigate the impact of Fru*f* side chains on the backbone conformation (PmE and PmB $\Delta$ Gly [Hie], Fig. 2, middle panel). Finally, the effect of the Gly amino acid side chain was established for PmB (Fig. 2, bottom panel).

## Materials and methods

We followed our established systematic approach to the modeling of polysaccharides as previously described (Richardson, Kuttel, et al. 2021; Richardson, Ravenscroft, et al. 2021; Richardson et al. 2022). For each of the 6 molecules of interest (Fig. 1), we built CPS chains of 3 RU and 6 RU.

Chain length is an important consideration when modeling CPSs, as a short chain may have insufficient molecular flexibility, while long chains are more computationally expensive to model. On the basis of our previous work, we consider a 6 RU chain to be representative of the behavior of the longer polysaccharide. Further, antibodies bind small fragments of the CPS between 1 and 7 residues in length (Kabat 1966) corresponding to between 1 and 4 RU in the case of *P. multocida*, making a 6 RU model sufficient to explore antibody Ep. We perform 3 RU simulations to ensure the behaviors of the short 3 RU and longer 6 RU chains are consistent, but here, we present only the 6 RU results (3 RU results are available in the supplementary information).

Molecular dynamics simulations in the aqueous solution for each of the chains followed a protocol of initial system equilibration (200 ns) and then a production run of 800–1,800 ns (3 RU simulations) or 1,300–2,300 ns (6 RU simulations), respectively, as required for convergence. Data analyses were performed on these simulation trajectories as described below.

### Molecular dynamics

The 3 RU and 6 RU chains were built using our CarbBuilder software (version 1.2.42) (Kuttel et al. 2011; Kuttel et al. 2016). Starting structures for each molecule were built using low-energy glycosidic linkage orientations obtained from potential mean force calculations (Supplementary Fig. S6) as per our previous work (Richardson, Ravenscroft, et al. 2021) The psfgen tool was used to create protein structure files (PSF) for simulation with the CHARMM36 additive force field for carbohydrates (Guvench et al. 2008, 2009). For the PmB molecule with Gly, we used the CHARMM36 protein force field parameters for Gly, which are compatible with the CHARMM36 additive force field (Guvench et al. 2011). We modeled only the 6 RU model for this molecule. Starting structures were minimized with the Nanoscale Molecular Dynamics (NAMD) program (version 2.13) for 10,000 steps at 300 K. Minimized structures were solvated using the Visual Molecular Dynamics (VMD) software (Humphrey et al. 1996) solvation and ionization tools to add cubic TIP3P (Jorgensen et al. 1983) water boxes of 50 Å per side for the 3 RU systems and 70 Å per side for the 6 RU systems. Systems were then neutralized with 1 sodium (Na^+^) counter ion per RU (3 ions for the 3 RU, 6 for the 6 RU). Initial minimization and heating protocols comprised 5 K incremental temperature reassignments beginning at 10 K up to 300 K with 500 steps of NAMD minimization and 8,000 steps of MD at each temperature reassignment. Solvated and ionized structures (Protein Data Bank and PSF files) for each 6 RU system are available as supplementary information.

Simulations of 3 RU and 6 RU were run using NAMD (version 2.13) (Phillips et al. 2005) with CUDA extensions for graphics processor acceleration (Stone et al. 2007).

Periodic boundary conditions equivalent to the cubic box size were employed for the solvated simulation with wrapping on. Long-range electrostatics were implemented with the Particle Mesh Ewald summation grid spacing set to 1 (Darden et al. 1993). Atoms were not held fixed, and the initial center of mass motion was turned off. The 1–3 pairs were excluded from nonbonded interactions, 1–4 interactions were not scaled, and the dielectric constant was set to 1. Smoothing functions were applied to both the electrostatics and van der Waal’s (VDW’s) forces with switching and cutoff distances of 10 Å and 12 Å, respectively.

A Leap-Frog Verlet integrator was used to integrate the equations of motion over a step size of 1 fs. A distance of 15 Å was used as the cutoff for inclusion in the pair list for the calculation of nonbonded forces. The short-range nonbonded interactions were calculated every 1 fs, full electrostatics calculations were performed every 2 fs, and atoms were reassigned every 10 fs (Van Gunsteren and Berendsen 1988).

Simulations were sampled under the isothermal-isobaric (nPT) ensemble. Langevin dynamics (Feller et al. 1995) were used to control the temperature with a damping coefficient of 5/ps. Nosé-Hoover Langevin piston dynamics were used as a barostat to maintain a target pressure of 1 atm (Nosé and Klein 1983; Hoover 1985). Variable system volume was used with a piston period of 100 fs and a decay of 50 fs. Simulations of 1,500 ns (2,500 ns for PmB $\Delta$ Gly and PmB) were performed for the 6 RU systems comprising 200 ns of equilibration and 1,300 ns (2,300 ns for PmB $\Delta$ Gly and PmB) of production run as was required for convergence (Supplementary Fig. S7). The 3 RU systems were run to 1,000 ns, except for PmE $\Delta$ Fru*f* which was run to 2,000 ns, as required for convergence (data available as Supplementary Figs. S1 and S8).

### Convergence

We addressed convergence using block standard averaging (Grossfield and Zuckerman 2009) applied to 2 metrics: end-to-end distance and radius of gyration (Supplementary Fig. S7). Block standard averaging was implemented with in-house Python scripts.

For all simulations, the blocked standard error reached plateaus for both metrics, indicating convergence. The simulation lengths were large multiples of the correlation times for end-to-end distance (PmE $\Delta$ Fru*f*, 6.3 ns; PmB $\Delta$ Fru*f*  $\Delta$ Gly, 9.24 ns; Hid $\Delta$  *X*, 5 ns; PmE, 1.4 ns; PmB $\Delta$ Gly, 47.7 ns; PmB, 101.63 ns) and radius of gyration (PmE $\Delta$ Fru*f*, 3.9 ns; PmB $\Delta$ Fru*f*  $\Delta$ Gly, 11.7 ns; Hid $\Delta$  *X*, 7.9 ns; PmE, 0.9 ns; PmB $\Delta$ Gly, 45.6 ns; PmB, 97.4 ns). Further, the numbers of independent samples were >>1 for both the end-to-end distance (PmE $\Delta$ Fru*f*, 237.8; PmB $\Delta$ Fru*f*  $\Delta$ Gly, 162.3; Hid $\Delta$  *X*, 299.2; PmE, 1067.7; PmB $\Delta$ Gly, 52.5; PmB, 24.6) and the radius of gyration (PmE $\Delta$ Fru*f*, 385.9; PmB $\Delta$ Fru*f*  $\Delta$ Gly, 127.7; Hid $\Delta$  *X*, 189.4; PmE, 1710.2; PmB $\Delta$ Gly, 54.9; PmB, 25.7). Our designated equilibration time of 200 ns is therefore greater than the correlation time.

### Data analysis

Molecular conformations were visualized using VMD, with the PaperChain and Twister visualization algorithms used to highlight carbohydrate rings and chains (Cross et al. 2009), as required.

Trajectories were extracted at 25-ps intervals with analysis performed on frames 250 ps apart. Metrics, such as end-to-end distances, were extracted from the simulation trajectories using Tcl scripting via VMD’s Tk console. Data analyses were performed with in-house Python scripts and plots were generated using Matplotlib (Hunter 2007).

#### Chain flexibility

The end-to-end distance, *r*, was measured from C1 of ManNAcA at the nonreducing end, to C3/4 of GlcNAc at the reducing end of each chain, thus excluding the highly flexible terminal residues.

#### Glycosidic linkages

Given the limited flexibility of the carbohydrate ring, the primary source of flexibility in the RU arises from the glycosidic linkages between neighboring monosaccharides. These glycosidic linkages are conveniently described by 2 dihedral angles, $\mathrm{\phi}$and $\mathrm{\psi}$. As per our previous work, we define $\mathrm{\phi}$ = H_1_-C_1_-O_1_-C’*_x_* and $\mathrm{\psi}$ = C_1_-O_1_-C’*_x_*-H’*_x_*, with *x* representing the linkage position (Richardson, Ravenscroft, et al. 2021). In the case of the Fru*f* linkages which are (2➔x)-linked, these definitions were adjusted to $\mathrm{\phi}$ = C_1_-C_2_-O_2_-C’*_x_* and $\mathrm{\psi}$ = C_2_-O_2_-C’*_x_*-H’*_x_*.

#### Conformational analysis

The most common chain conformations for each simulation were determined by clustering the production trajectory frames into families and by calculating the relative occupancies of each family. Clusters comprising <6% of the production run (post equilibration) were discarded. Clustering was performed using the WMC PhysBio plug-in for VMD’s built-in measure cluster command (Luis 2012). Prior to clustering, the molecules were aligned on the RU 3 and RU 4 residues excluding the Fru*f*/Gly residue(s) and any hydrogens on the backbone. Clustering was then performed as an RMSD fit to the ring and linkage atoms of the central 4 RUs of the chains excluding nonring atoms, the Fru*f*/Gly residue(s), and the highly flexible terminal RU 1 and RU 6. Five clusters were created with a cutoff of 3 Å.

Clustering analysis was also performed on the central 2 RUs, RU 3 and RU 4. Alignment was performed using the ManNAcA residue of RU 4 (excluding hydrogens) and was followed by clustering using the ring and linkage atoms of RU 3 and RU 4 excluding nonring atoms and Fru*f*/Gly residue(s), creating 3 clusters with a cutoff of 1.1 Å, before discarding clusters comprising <6% of the trajectory.

#### Solvent accessible surface

Hydrophilic/hydrophobic regions of the molecular surface were analyzed using VMD’s built in “measure sasa” command. The solvent accessible surface area (sasa) analysis was performed by probing first hydrophilic regions (comprising hydroxyl groups, carbonyl groups, ring oxygens, nitrogen, and linkage oxygens) and then hydrophobic/neutral regions (comprising methyl groups, CH_2_ groups, ring carbons, and ring protons) of the molecule using a VDW’s radius of 2.5 Å—larger than that of water to imitate a potential small binding molecule. The ratio of hydrophilic sasa to total sasa was then calculated to determine the percentages of the hydrophilic and hydrophobic surface area exposed to solvent.

## Results

We compare the effect of differing backbone linkages and substituents on the conformation and dynamics of the 6 CPS chains. We begin with a comparison of CPS chain extension and flexibility, followed by the molecular conformations, and finally analyze the potential binding epitopes (Ep) exposed on the saccharide chains.

### Chain extension and flexibility

**Fig. 3:**
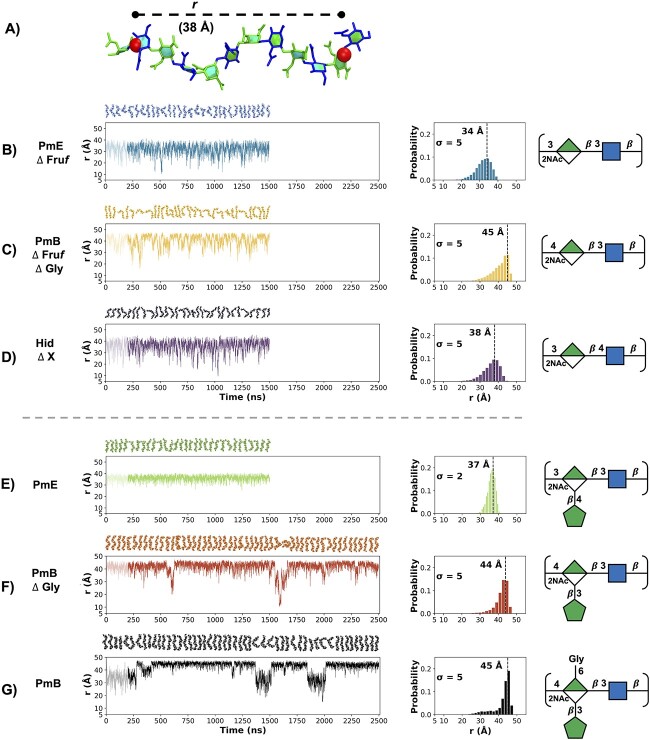
Time series of the end-to-end distance, *r*, in the 6 RU CPS chains. A) *r* is measured (Å) from C3/C4 of GlcNAc at the reducing end to C1 of ManNAcA at the nonreducing end, thus excluding the highly flexible terminal residues. Time series plots (left column) and corresponding histograms (center column) for the simulation trajectories are shown for: B) PmE $\Delta$ Fru*f*, C) PmB $\Delta$Fru*f*  $\Delta$ Gly, D) Hid$\Delta$  *X*, E) PmE, F) PmB $\Delta$Gly, and G) PmB; the initial 200 ns (indicated by lighter shade) are considered initial equilibration and the remaining trajectory is the production run. *X* represents a variable amino acid moiety: L-alanine, L-serine, or L-threonine. Conformational snapshots at 50-ns intervals are shown above the time series plots; SNFG line structures are shown in the right column. The histograms are labeled with the standard deviations ($\mathrm{\sigma}$) and modal peak *r* value.

The molecular extension and flexibility of a carbohydrate is commonly measured by the fluctuation in the end-to-end distance, *r*, of the chain, during a simulation (Fig. 3A). Times series plots of *r* for the simulations of 6 RUs of each of the 6 CPSs reveal considerable differences in the chain extension and dynamics across the antigens (Fig. 3B–G, left column). This is consistent with the trend observed for the 3 RU chains (Supplementary Fig. S1).

The 3 unbranched CPS chains (Fig. 3B–D) have similar flexibilities ($\mathrm{\sigma}$ = 5 for all 3 6 RU molecules), with a wide range of conformations (see the trajectory snapshots at 50-ns intervals shown above the graphs). However, the backbones show considerably different chain extensions, as is clear from the *r* distribution histograms (Fig. 3, center column). PmE $\Delta$ Fru*f,* comprising all $\mathrm{\beta}$(1→3) glycosidic linkages, is the least extended chain, with a modal *r* value of 34 Å (Fig. 3B). Replacement of either of the 2 glycosidic linkages in the backbone with a $\mathrm{\beta}$(1→4) linkage increases the length of the RU and thus the overall chain extension: Hid $\Delta$  *X* (with a 4-linked GlcNAc) has an increased modal *r* value of 38 Å (Fig. 3D), and PmB $\Delta$ Fru*f*  $\Delta$ Gly (with a 4-linked ManNAcA) is the most extended chain with a modal *r* value of 45 Å (Fig. 3C).

Addition of Fru*f* and/or Gly side chains to the ManNAcA backbone residue has little effect on the CPS chain extension, the modal *r* values remain very similar to the unsubstituted backbones: PmE 37 Å (Fig. 3E), PmB $\Delta$ Gly 44 Å (Fig. 3F) and PmB 45 Å (Fig. 3G). Interestingly, side chains considerably decrease the flexibility of the $\mathrm{\beta}$(1→3)-linked PmE ($\mathrm{\sigma}$ = 2), but not the $\mathrm{\beta}$(1→4)-linked PmB molecules ($\mathrm{\sigma}$ = 5 for both). The decrease in PmE flexibility can be explained by the increased steric hindrance: The addition of a side chain to the already highly substituted ManNAcA residue (2-NAc, 5-COOH substituted, and 3- or 4-linked to neighbor) results in all ring hydroxyls being substituted. Close comparison of the *r* time series plots in Fig. 3C (PmB $\Delta$ Fru*f*  $\Delta$ Gly) with Fig. 3F (PmB $\Delta$ Gly) and G (PmB) shows a similar decreased flexibility of the chain on a ~250-ns time scale. However, on a 1,000-ns time scale, new populations of very low *r* values appear, which maintain the overall chain flexibility on a level similar to the unsubstituted backbone. Interestingly, in the more substituted PmB, these populations occur more frequently than in PmB $\Delta$ Gly.

The glycosidic linkages are the primary source of flexibility in the CPS chains. Side chains on ManNAcA reduce the overall flexibility of the chains through impeding rotation of the ManNAcA→GlcNAc glycosidic linkage through steric clashes between neighboring residues; rotation of the GlcNAc→ManNAcA is largely unaffected (Supplementary Fig. S2 and Supplementary Table S1).

### CPS chain conformations

**Fig. 4:**
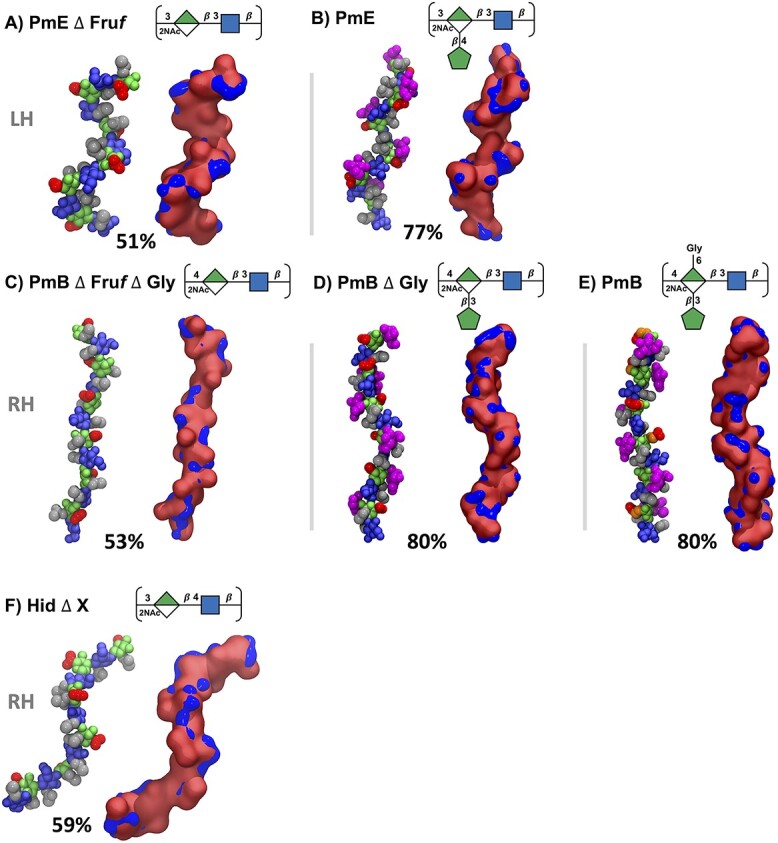
Primary conformational families determined by cluster analysis of RU 2 to RU 5 of the CPS for A) PmE $\Delta$Fru*f*, B) PmE, C) PmB $\Delta$Fru*f*  $\Delta$Gly, D) PmB $\Delta$Gly, E) PmB, and F) Hid$\Delta$  *X*. The handedness of the helix is indicated on the left of the figure by left-handed (LH) or right-handed (RH). *X* represents a variable amino acid moiety: L-alanine, L-serine, or L-threonine. Conformations are visualized with the VDW (left) and Quicksurf representations (right). VDW representations were colored as for SNFG symbols: ManNAcA, green; GlcNAc, blue; NAc, gray; COOH, red; Fru*f*, magenta; and Gly, orange. For the Quicksurf representation, hydrophobic and neutral atoms were colored red, and hydrophilic atoms were colored blue.

For all the 6 RU CPSs, the primary conformations are well-defined helices (Fig. 4), which dominate for >50% of simulation time in all cases (minor conformational clusters and the cluster occupancy are shown in the Supplementary Figs. S3 and S4), which is consistent with the conformations observed in the 3 RU chains (Supplementary Fig. S1). The helices, however, differ in their handedness according to the constituent glycosidic linkages: the $\mathrm{\beta}$(1→3)-linked PmE (Fig. 4A) and the PmE $\Delta$ Fru*f* unsubstituted backbone (Fig. 4B) form right-handed helices, whereas the molecules containing a $\mathrm{\beta}$(1→4) glycosidic linkage—PmB $\Delta$ Fru*f*  $\Delta$ Gly (Fig. 4C), PmB $\Delta$ Gly (Fig. 4D), PmB (Fig. 4E), and Hid $\Delta$  *X* (Fig. 4F)—all form left-handed helices. Further, the PmE molecules are more compact than the PmB molecules.

In all cases, addition of the Fru*f* and/or Gly side chains reduces the flexibility of the CPS backbone. For PmE, a Fru*f* side chain increases the occupancy of the primary helical conformation from 51% (PmE $\Delta$ Fru*f*, Fig. 4A) to 77% (PmE, Fig. 4B) and results in a more extended helix; the modal *r* distance increases from 34 Å to 37 Å (Fig. 3). Similarly, the 53% occupancy of the main helical conformation of PmB $\Delta$ Fru*f*  $\Delta$ Gly (Fig. 4C) increases to 80% in PmB $\Delta$ Gly (Fig. 4D), although in this case, the helices have similar extensions. The addition of a Gly side chain in PmB (Fig. 4E) does not alter the backbone conformation or flexibility of the primary helical conformation. Further, the Fru*f* (Fig. 4B, D, and E, magenta) or Gly (Fig. 4E, orange) side chains are highly solvent-exposed and thus present potential targets for antibody binding while also shielding the backbone residues.

**Fig. 5:**
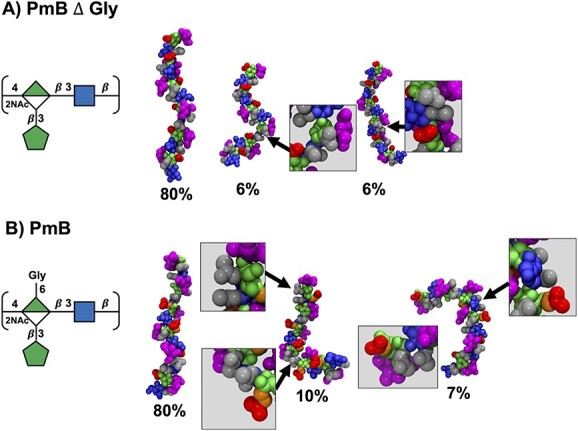
The main conformational families of A) PmB $\Delta$Gly and B) PmB with NAc pairing of the nonprimary clusters indicated in zoomed in boxes. The VDW’s representation was used with colors as per SNFG: ManNAcA residues are shown in green, GlcNAc residues in blue, NAc groups in gray, COOH groups in red, Fru*f* in magenta, and Gly in orange.

The alignment of the hydrophobic (NAc, gray) and hydrophilic (COOH, red) residue substitutions differs in the 3 backbones, resulting in a considerable difference in surface hydrophilicity (Supplementary Fig. S5). For PmE $\Delta$ Fru*f* (Fig. 5A), the NAc and COOH moieties are well separated on the backbone, whereas PmB $\Delta$ Fru*f*  $\Delta$ Gly has frequent shielding of the groups of ManNAcA COOH by the NAc on an adjacent GlcNAc residue, resulting in a considerably less hydrophilic surface. However, the Fru*f* side chains with their many exposed hydroxyl groups increase the hydrophilicity of the molecular surface (Fig. 5, Quicksurf visualizations). This is particularly in the case of PmB (Supplementary Fig. S5), which has the potential to impact antibody binding.

**Fig. 6:**
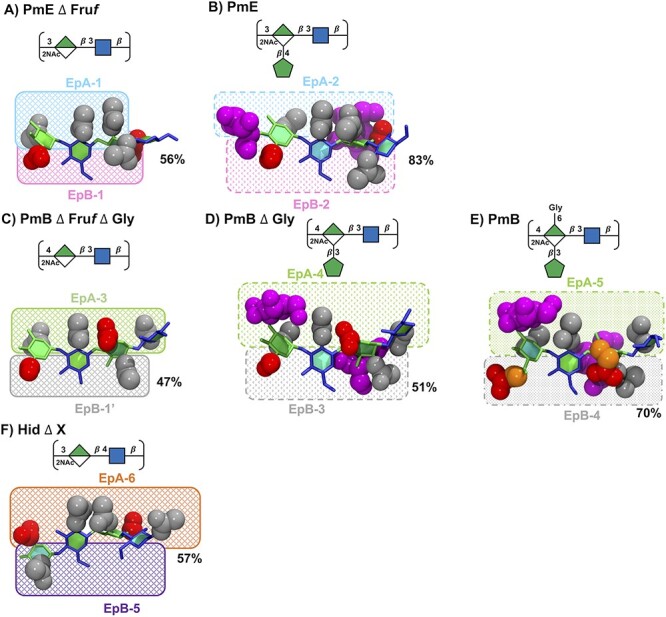
Main conformations and associated percentages identified from clustering of RU 3 and RU 4 only with conformational epitopes (Ep) indicated by shaded boxes for: A) PmE $\Delta$Fru*f*, B) PmE, C) PmB $\Delta$Fru*f*  $\Delta$Gly, D) PmB $\Delta$Gly, E) PmB, and F) Hid $\Delta$*X*. *X* represents a variable amino acid moiety: L-alanine, L-serine, or L-threonine. The VDW’s representation was used with colors as per SNFG: ManNAcA residues are shown in green, GlcNAc residues in blue, NAc groups in gray, COOH groups in red, Fru*f* in magenta, and Gly in orange.

As discussed above, the simulations of PmB $\Delta$ Gly and PmB show minor populations with short *r* distances. These populations are associated with bent conformations of the CPS chain. The bent conformations align adjacent NAc groups (Fig. 6A, E, and F), which may assist with conformational stabilization.

### Epitopes

As the CPS regions bound by antibodies comprise 1–7 residues, it is useful to compare the 6 RU CPS chains on this length scale. Figure 6 focuses on the principal conformations of the 2 central repeat units (RU 3 and RU 4) for each of the 6 molecules; potential Ep are indicated by shaded boxes. There are key differences in the alignment of the side chains and residue substitutions, forming a range of different epitopes (despite a relatively conserved backbone). In the following analysis, we focus on substitutions on opposing sides of the CPS chain, which we term as A and B.

For side A, the PmE $\Delta$ Fru*f* molecule (Fig. 6A) has a hydrophobic epitope exposing 3 partially aligned N-acetyl groups, EpA-1. On side B, EpB-1 comprises the exposed COOH (red), of C6 on ManNAcA, and the N-acetyl on GlcNAc in the following RU. In PmE, the Fru*f* substitution considerably changes side A, forming the EpA-2 epitope with the 3 aligned NAc groups seen in EpA-1, now shielded by the Fru*f* residue. Similarly, on side B, the Fru*f* shields the exposed COOH forming the EpB-2 epitope (the NAc would also be shielded by the adjacent Fru*f* as in PmE the Fru*f* residues are on alternate faces). Conformationally, we see an increased conformational definition as the occupancy increases from 56% in PmE $\Delta$ Fru*f* to 83% in PmE.

In PmB $\Delta$ Fru*f*  $\Delta$ Gly (Fig. 6C), side A of the chain is considerably altered (compared to PmE $\Delta$ Fru*f*), with 1 NAc replaced by a hydrophilic carboxylic acid group and a further NAc joining the group, forming EpA-3. Side B remains similar to the PmE molecules, though, with the ManANAcA N-acetyl replacing the GlcNAc N-Acetyl—forming EpB-1′. Fru*f* substitution in PmB $\Delta$ Gly (Fig. 6E) completely disrupts the molecular surface presented on both sides of the CPS with shielding Fru*f* residues forming epitopes EpA-4 and EpB-3. Conformationally, we see the occupancy increase from 47% to 51%. Additional Gly substitution in PmB (Fig. 6F) replaces COOH groups with bulkier Gly and thus further alters both sides of the chain, forming epitopes EpA-5 and EpB-4. The addition of Gly further increases the occupancy to 70% and suggests that for this longer, less compact, helix, an additional side group is required to achieve adequate shielding.

Interestingly, Hid $\Delta$  *X* (Fig. 6C) has altered both sides of the chain, forming EpA-6 and EpB-5. On side A, the NAc pairs are sandwiched by COOH groups on either side with ¼–½ turn between NAc and COOH groups; on the mostly unsubstituted side B, the NAc of the ManNAcA is positioned where the COOH is in PmE and PmB. The central paired NAc in EpA-6 may be similar enough to EPA-1 to allow for antibody cross-reaction.

## Discussion

This study of 6 structurally similar CPS provides further evidence that small structural differences in a carbohydrate chain can have a large impact on both molecular conformation and the epitopes exposed for antibody binding. Although *P. multocida* types E and B (PmE and PmB) as well as the related Hie and Hid CPS molecules all have well-defined helical molecular conformations, the presence of $\mathrm{\beta}$(1→4) glycosidic linkages in the backbone changes both the handedness of the helix and increases the extension of the helices relative to chains with only $\mathrm{\beta}$(1→3) linkages. Further, side chain substitution of the backbone with Fru*f* decreases the molecular flexibility, with a concomitant increase in conformational definition. This has the potential to increase the antigenicity of the CPS fragments, as a more conformationally defined molecule presents a better target for antibodies. However, side chain substitutions also considerably alter the Ep and potentially shield the ManNAcA-GlcNAc backbone from antibody binding with the longer, more accessible serotype B helices, requiring >1 side group to achieve the same level of backbone shielding as those of serotype E. The ManNAcA and GlcNAc amino sugars are expected to be immunogenic. The ManNAcA residue is often found with other amino sugars and is a motif commonly expressed by pathogens (Adlam et al. 1984; Yoneyama et al. 1984; Acker and Kammerer 1990). Further, mannose-binding proteins found in the lungs and serum of *Bovidae* (such as surfactant proteins, mannose-binding proteins, and collectins) are highly specific for fucose, mannose, N-acetyl-D-mannosamine, N-acetyl-D-glucosamine, and glucose, playing an important role in immune activation and bacterial clearance (Kawasaki et al. 1985; Holmskov et al. 1993; Neth et al. 2000). Therefore, we expect that the highly exposed Fru*f* and Gly substituents may be used to disguise an immunogenic backbone.

Our simulations suggest very limited cross-reactivity between any of the 6 CPSs we modeled, which is in line with the immunological data currently available. Early studies using whole-cell killed vaccines as well as CPS extracts demonstrated limited cross-protection between serotypes B and E (Penn and Nagy 1974, 1976; Nagy and Penn 1976). Elucidation of the CPS structures containing the acid-labile Fru*f* (St Michael et al. 2021) allows interpretation of these experiments as well as the systematic modeling studies presented here. The significant differences that we have found in the CPS backbone conformations and lack of common epitopes between the CPS predict limited cross-reactivity and cross-protection of these 2 serotypes, therefore supporting the development of a bivalent CPS-based vaccine. A bivalent vaccine has been found to be effective against a type E challenge in cattle with passive mice protection against both types E and B, suggesting the cattle should also be protected against type B (Nagy and Penn 1976).

For the closely related Hie and Hid CPSs, we also suggest a lack of cross-reactivity as the Hid $\Delta$  *X* CPS has very different conformations and potential Ep to the Hie (PmB $\Delta$Gly) and, most notably, the absence of the Fru*f* residue in Hid has an impact on the binding surface of the molecule as well as on the potential epitopes. This may also account for Hid being a less successful pathogen: The lack of Fru*f* side chain results in more exposure of the immunogenic backbone. Low levels of cross-reactivity between Hie and Hid may occur with limited cross-protection. Although the variable amino acid substituents of Hid (L-alanine, L-serine, and L-threonine) were not modeled here, we expect a similar result with the amino acids having little impact on conformation but shielding the backbone although potentially less effectively than Fru*f*.

Our modeling investigation of *Streptococcus pneumoniae* serogroup 10 found that strongly cross-reactive serotypes share common epitopes (Richardson et al. 2022). Here, we find no evidence for common epitopes between the 6 CPSs studied, which suggest little potential for cross-protection between them. This is supported by early immunological studies with PmE and PmB CPSs that showed low levels of limited cross-protection. However, direct structural information on the interactions of CPSs with antibodies is required for corroboration of our prediction that the lack of cross-protection between the CPS is due to an absence of shared epitopes. Unfortunately, molecular binding studies of CPSs with bactericidal antibodies are not currently available and are rarely feasible. In the absence of direct structural data, immunological studies using reciprocal PmB and PmE CPS (or, preferably, the CPS conjugate vaccines and structural mutants modeled here) would assist in determining the importance of specific structural moieties/epitopes on the CPS for cross-protection. Such validation would be extremely valuable, as elucidation of the structural basis for cross-protection would greatly aid in the rational development of more effective CPS-based vaccines against *P. multocida*.

## Supplementary Material

Richardson_Ravenscroft_Kuttel_2023_Supplementary_revision_cwad049

## Data Availability

The data underlying this article are available in the article and in its online supplementary material, and any other data will be shared upon reasonable request to the corresponding author.

## CRediT authors’ statement

Nicole I. Richardson (Investigation [equal], Validation [lead], Visualization [lead], Writing—original draft [lead]), Neil Ravenscroft (Conceptualization [equal], Funding acquisition [equal], Investigation [equal], Methodology-Supporting, Project administration [equal], Resources [equal], Supervision [equal], Writing—review & editing [equal]), and Michelle M. Kuttel (Conceptualization [lead], Data curation [equal], Funding acquisition [equal], Investigation [equal], Methodology [equal], Project administration [equal], Software [equal], Supervision [equal], Validation [equal], Visualization-Supporting, Writing—review & editing [equal])

## Abbreviations

COOH carboxylic acid

CPS capsular polysaccharide

Ep epitope

Fru*f*  $\mathrm{\beta}$-D-fructofuranose

GlcNAc $\beta$-D-glucosamine

Gly glycine

Hid *H. influenzae* type d

Hie *H. influenzae* type e

ManNAcA N-acetyl-$\mathrm{\beta}$-D-mannosaminuronic acid

NAc N-acetyl

NAMD Nanoscale Molecular Dynamics

PmB *P. multocida* type B

PmE *P. multocida* type E

PSF protein structure file

RU repeat unit sasa solvent accessible surface area

SNFG Symbol Nomenclature for Glycans

VMD Visual Molecular Dynamics

VDW van der Waal
