# Supplementary material for: Conformational comparisons of Pasteurella multocida types B and E and structurally related capsular polysaccharides
Source: Glycobiology. 2023 Jun 19;33(9):745–54. doi: 10.1093/glycob/cwad049 (PMC10627249; doi:10.1093/glycob/cwad049)
Supplement: Richardson_Ravenscroft_Kuttel_2023_Supplementary_revision_cwad049 [file richardson_ravenscroft_kuttel_2023_supplementary_revision_cwad049.docx]

**Supplementary Information**

| 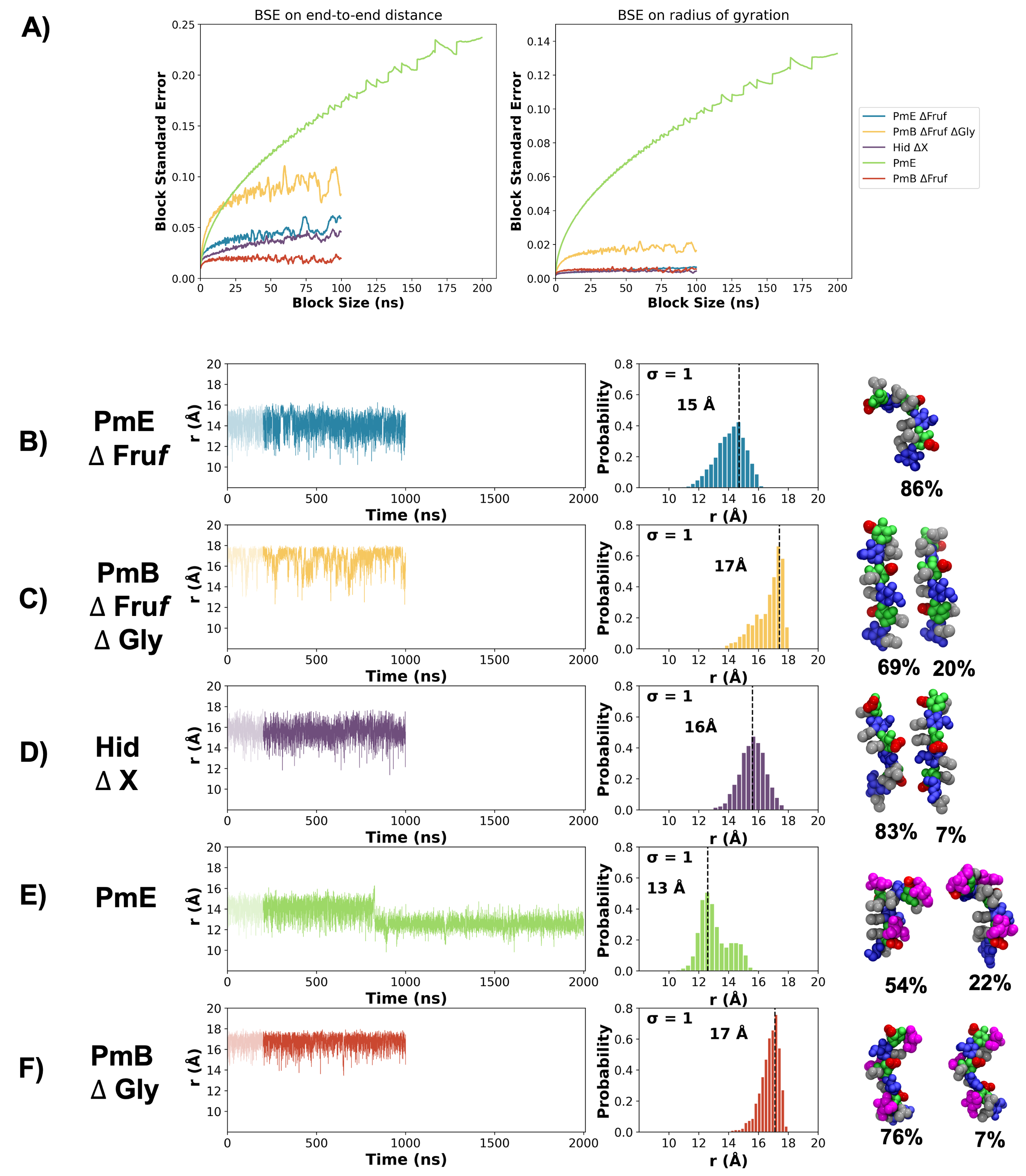  **Figure S 1:** (A) Block standard averaging analysis for modeled 3 RU P. multocida and H. influenzae CPS molecules with block standard error (BSE) versus block size (ns) calculated on end-to-end distance in the left column and BSE versus block size calculated on radius of gyration in the right column. For all molecules, the BSE visually reaches a plateau. Time series of the end-to-end distance, r, in the 3 RU CPS chains with r defined as the distance (Å) from C3/C4 of GlcNAc of RU 1 to C1 of ManNAcA in RU 3. Time series plots (left column) and corresponding histograms (right column) for the simulation trajectories as well as main clusters are shown for: (B) PmE $\Delta$ Fruf, (C) PmB $\Delta$Fruf, (D) Hid $\Delta$ X, (E) PmE, and (F) PmB $\Delta$ Gly. X represents a variable amino acid moiety: L-alanine, L-serine, or L-threonine. For each trajectory the initial 200 ns (lighter coloring) are considered initial equilibration and the remaining trajectory is the production run. The histograms are labelled with the standard deviations ($\sigma$) and modal peak r value. For the clustering analysis, alignment was performed on the central (RU 2) backbone residues (excluding Fruf) and clustering was performed on the ring and backbone atoms excluding any side groups with five clusters and a cutoff of 1.5 Å. Clusters are shown in the VDW (van der Waal) representation using colors as per SNFG were used (Haltiwanger 2016; Neelamegham et al. 2019; Varki et al. 2015): ManNAcA residues are shown in green, GlcNAC residues in blue, NAC groups in grey, COOH groups in red, and Fruf in magenta. Notably PmE shows two distinct populations of r which correspond to different alignments of NAc groups (grey) in the molecule. The primary occupancy at 54 % resembles a minor cluster in the 6 RU molecule and is highly prevalent largely due to the short nature of the chain and the high flexibility of the terminal repeating units. |
| --- |
|  |

| 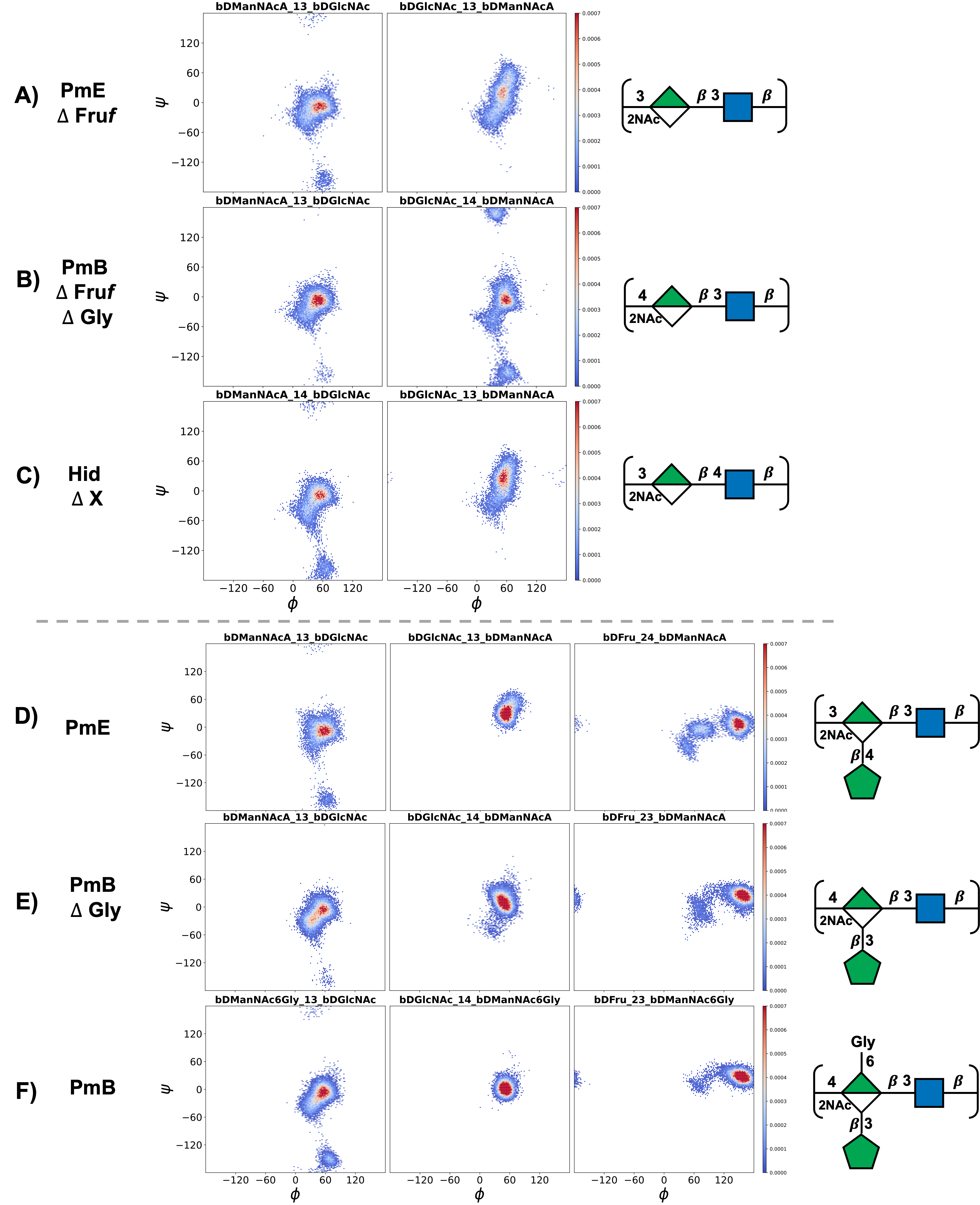  **Figure S 2:** Heatmap plots for the modeled 6 RU molecules showing phi ($\phi$) vs psi ($\psi$) dihedral angles for ManNAcA🡪GlcNAc and GlcNAc🡪ManNAcA glycosidic linkages of: (A) PmE $\Delta$ Fruf, (B) PmB $\Delta$Fruf $\Delta$Gly, and (C) Hid $\Delta$X as well as for the ManNAcA🡪GlcNAc and GlcNAc🡪ManNAcA and Fruf🡪ManNAcA linkages of (D) PmE, (E) PmB $\Delta$ Gly, and (F) PmB. X represents a variable amino acid moiety: L-alanine, L-serine, or L-threonine. The linkages were defined as we have done previously for these types of linkages (Richardson et al. 2022; Richardson et al. 2021). Glycosidic linkages were defined as φ = H_1_-C_1_-O_1_-C’_x_ and ψ = C_1_-O_1_-C’_x_-H’_x_. For the heatmaps, higher occupancy is indicated by redder areas and lower occupancy by bluer areas as shown in the legend on the right of the heatmaps. SNFG line structures are shown in the right column for each molecule. The backbone molecules (A, B, and C) have similar major occupancies (represented by redder areas on the heatmap) with the PmB $\Delta$ Fruf $\Delta$ Gly (4-linked ManNAcA) more flexible in the GlcNAc🡪ManNAcA linkage and Hid $\Delta$ X (4-linked GlcNAc) in the ManNAcA🡪GlcNAc linkage than PmE $\Delta$ Fruf (fully 3-linked backbone). The substituted molecules (D, E, and F) have similar primary occupancy, but are less disperse and flexible than the backbone molecules (redder and less disperse heatmaps) which is expected from the steric hindrance caused by greater substitution. Further, the Fruf🡪ManNAcA linkages are very similar in primary occupancy, but for PmE this linkage is more disperse and flexible than for PmB $\Delta$ Gly or PmB. |
| --- |

**Table S I:** Tabulation of the phi, psi ($\phi$,$\psi$) dihedral angles for each glycosidic linkage of the modeled molecules.

| **Molecule** | **ManNAcA 🡪 GlcNAc**  **(**$\boldsymbol{\phi}$**,**$\boldsymbol{\psi}$**)** | **GlcNAc 🡪 ManNAcA**  **(**$\boldsymbol{\phi}$**,**$\boldsymbol{\psi}$**)** | **Fruf 🡪 ManNAcA**  **(**$\boldsymbol{\phi}$**,**$\boldsymbol{\psi}$**)** |
| --- | --- | --- | --- |
| **PmE** $\Delta$ **Fruf** | 45, -10 | 45, 40 | N/A |
| **PmB** $\Delta$ **Fruf** $\Delta$ **Gly** | 45, -10 | 60, -10 | N/A |
| **Hid** $\Delta$ **X** | 45, -10 | 45, 60 | N/A |
| **PmE** | 50, -10 | 45, 30 | 150, 10 |
| **PmB** $\Delta$ **Gly** | 50, -5 | 40, 15 | 165, 25 |
| **PmB** | 50, -10 | 45, 0 | 160, 25 |

| 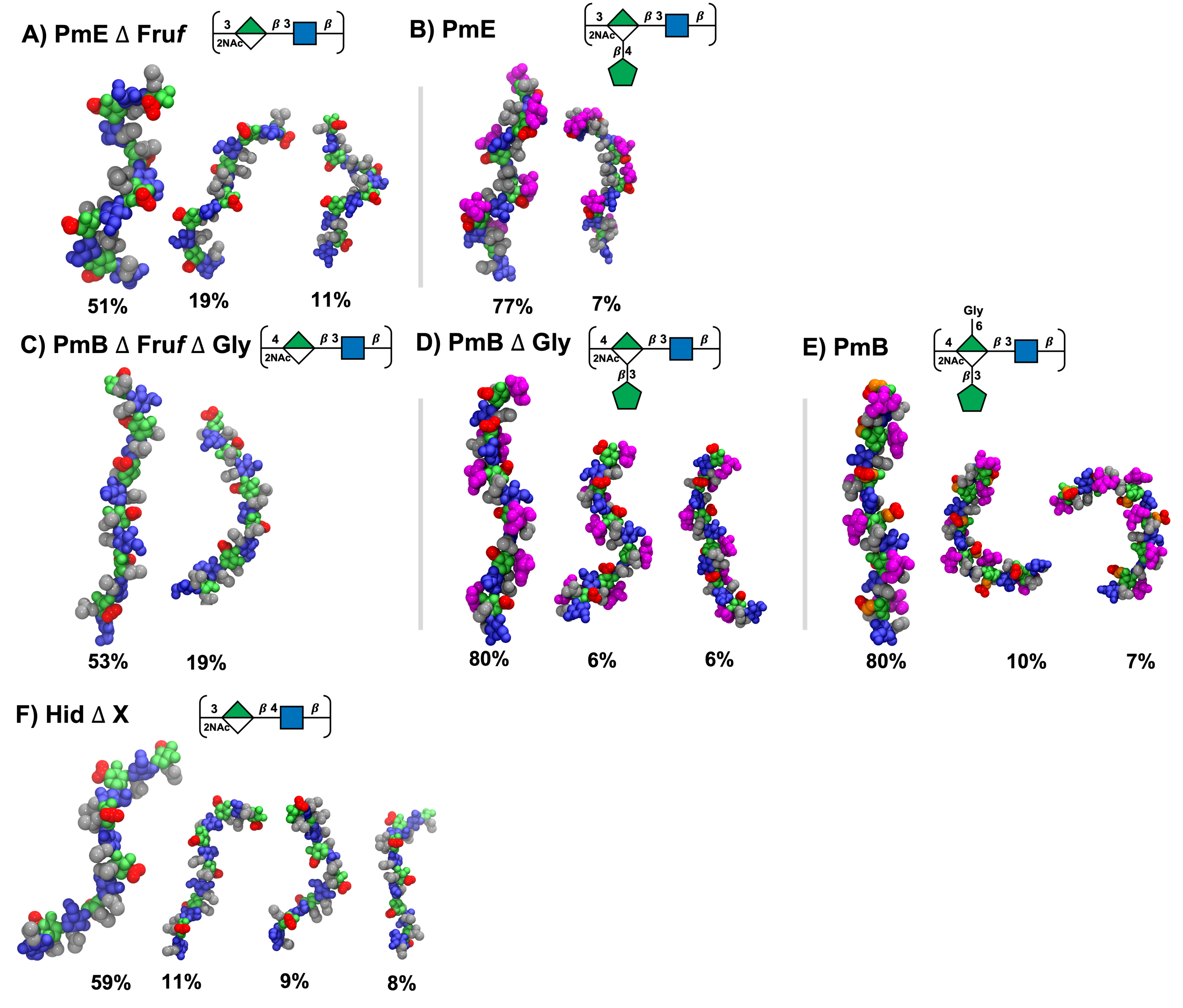  **Figure S 3**: Main conformational families and associated percentages identified for the modeled 6 RU P. multocida CPS molecules with the terminal repeat units (RU 1 and RU 6) excluded from analysis. (A) PmE $\Delta$ Fruf, (B) PmE, (C) PmB $\Delta$ Fruf, (D) PmB $\Delta$ Fruf $\Delta$ Gly, (E) PmB, and (F) Hid $\Delta$ X. X represents a variable amino acid moiety: L-alanine, L-serine, or L-threonine. The VDW (van der Waal) representation was used with colors as per SNFG: ManNAcA residues are shown in green, GlcNAc residues in blue, NAc groups in grey, COOH groups in red, Fruf in magenta, and Gly in orange. Line structures are also shown above the clusters using SNFG colors. |
| --- |

| 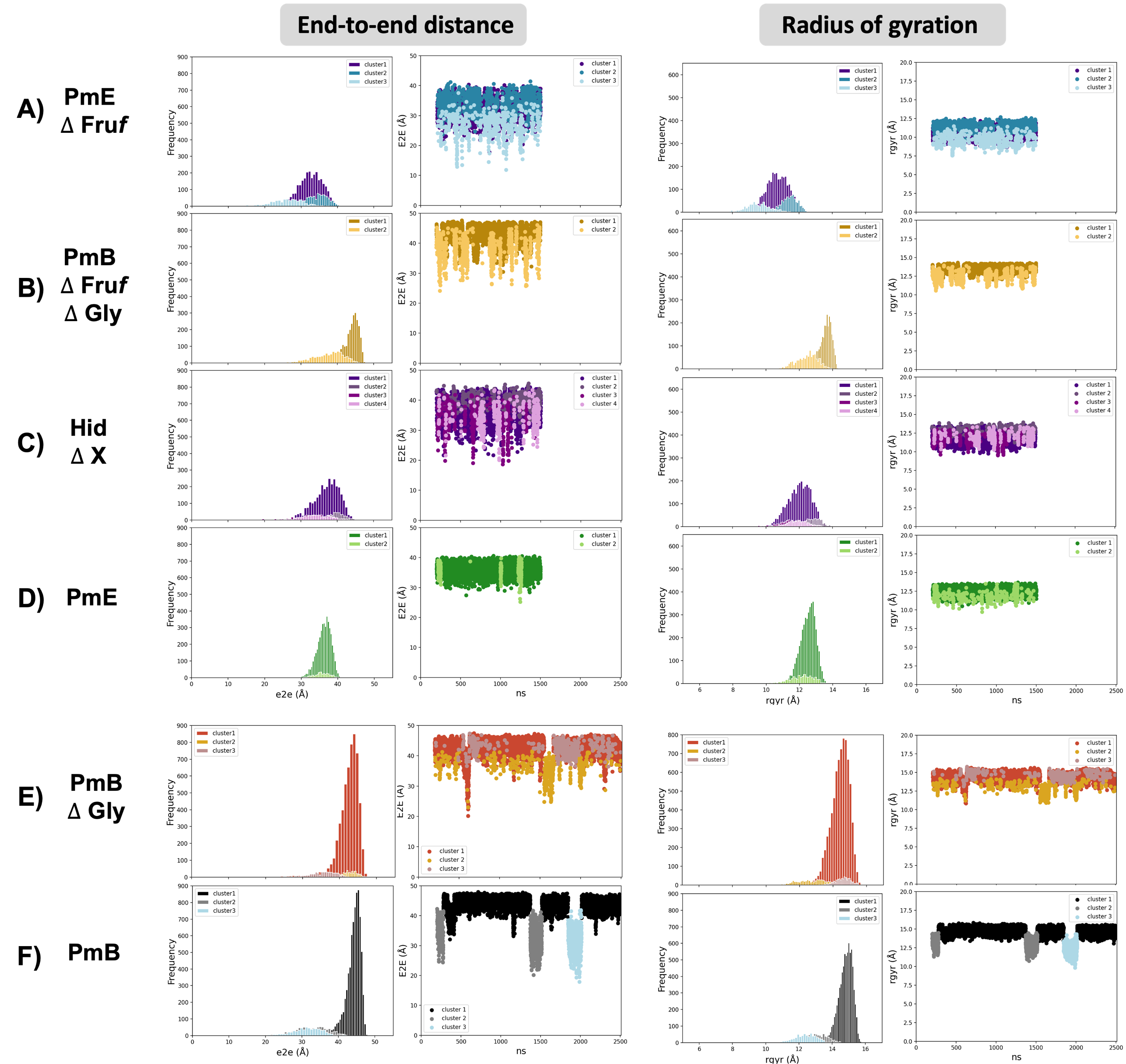  **Figure S 4**: Distribution and time series plots of the end-to-end distance and radius of gyration versus time (ns) for the main conformational families of the modeled 6 RU molecules: (A) PmE $\Delta$Fruf, (B) PmB$\Delta$Fruf $\Delta$Gly, (C) Hid $\Delta$X, (D) PmE, (E)PmB $\Delta$Gly, and (F) PmB. X represents a variable amino acid moiety: L-alanine, L-serine, or L-threonine. |
| --- |


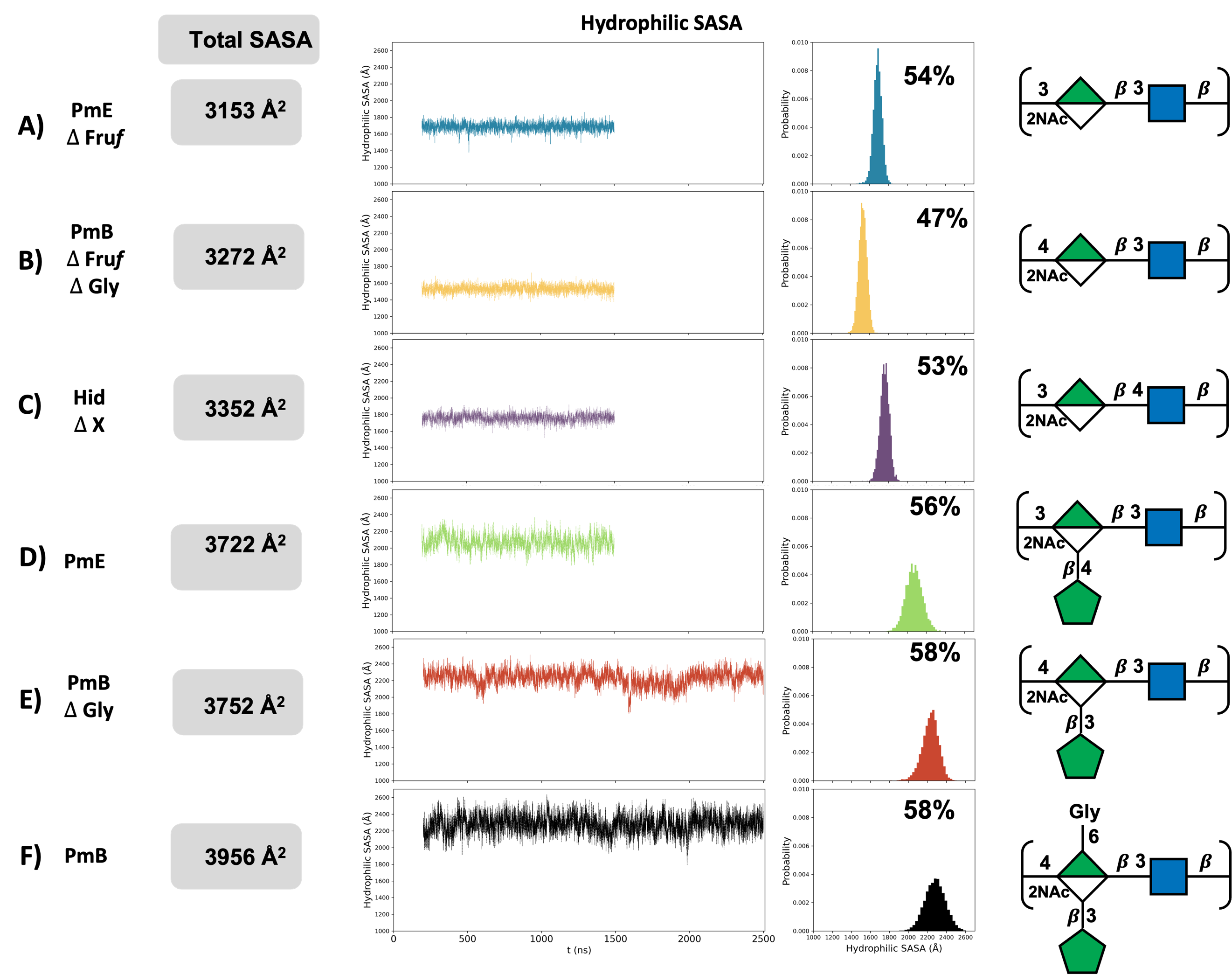


**Figure S 5**: Total solvent accessible surface area (sasa) to a 2.5 Å probe (left column) with time series plots of hydrophilic sasa (Å) vs time (ns) (middle column) and histograms showing probability distributions of hydrophilic sasa (Å) (right column) for the following 6 RU molecules: (A) PmE $\Delta$Fruf, (B) PmB $\Delta$Fruf $\Delta$ Gly, (C) Hid $\Delta$X, (D) PmE, (E)PmB $\Delta$Gly, and (F) PmB. X represents a variable amino acid moiety: L-alanine, L-serine, or L-threonine. Average percentage of the surface area that is hydrophilic is indicated on the histograms in the right column. The backbones have similar total sasa increasing in the order: PmE ∆ Fruf < PmB ∆ Fruf ∆ Gly < Hid ∆ X. The hydrophilic sasa, however, has a different order: PmB ∆ Fruf ∆ Gly < Hid ∆ X < PmE ∆ Fruf. Thus, adding Fruf increases both the total sasa and the hydrophilic sasa for PmE (54 % to 56 %) and PmB ∆ Gly (47 % to 58 %) with the greater increase for the PmB molecule potentially due to greater extension in this molecule. The subsequent addition of Gly to PmB again increases the total surface area, but the relative hydrophilic surface stays the same (58 %).


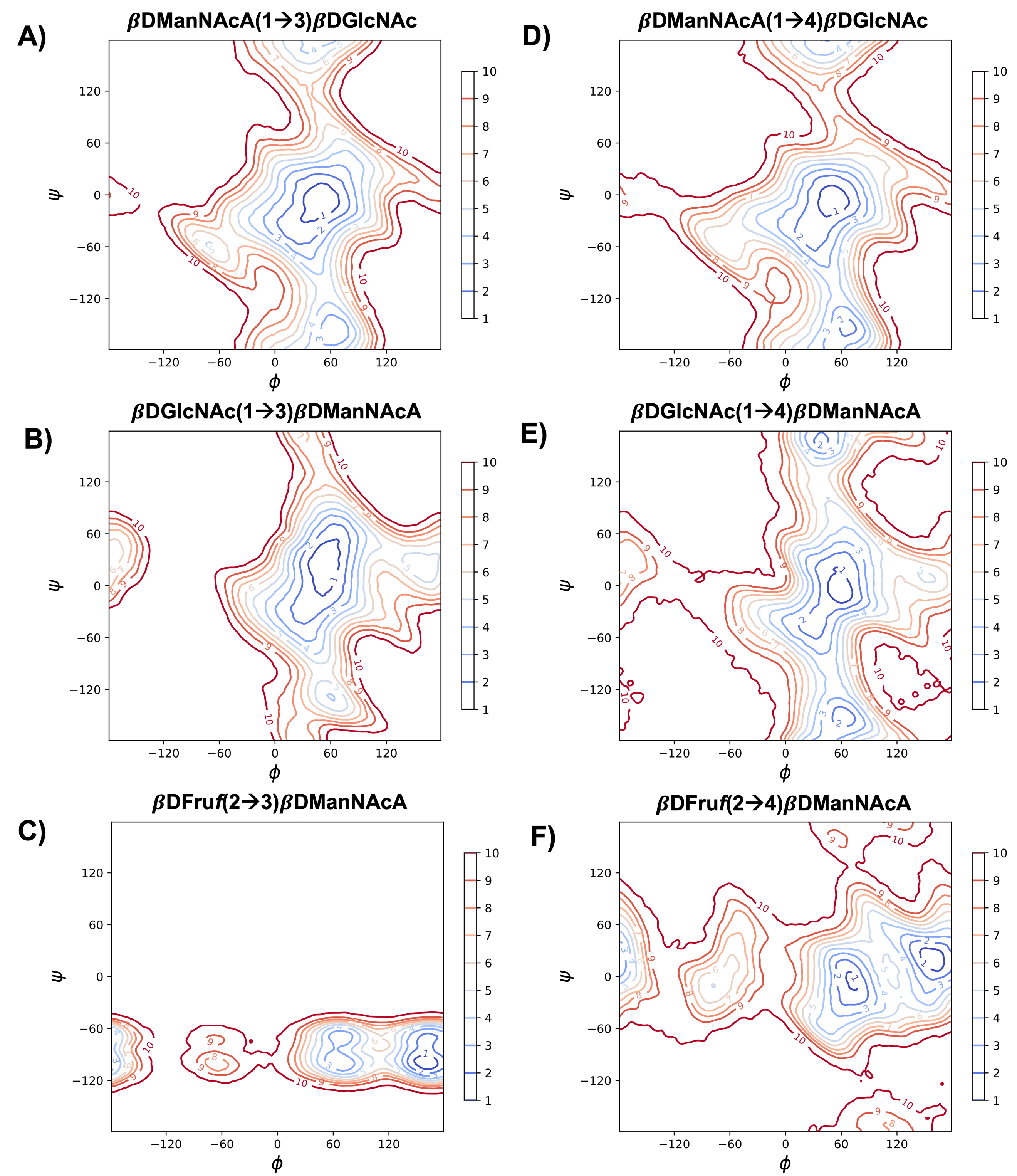


**Figure S 6**: Contour plots for the PMF calculations of the disaccharide glycosidic linkages for the modeled molecules. The left column displays the 1🡪3 linked PMFs for: (A) ManNAcA(1🡪3)GlcNAc, (B) GlcNAc(1🡪3)ManNAcA, (C) Fruf(2🡪3)ManNAcA. The right column displays the 1🡪4 linked PMFs for: (D) ManNAcA(1🡪4)GlcNAc, (E) GlcNAc(1🡪4)ManNAcA, (F) Fruf(2🡪4)ManNAcA. Higher energy is represented by redder lines and lower energy by bluer lines.


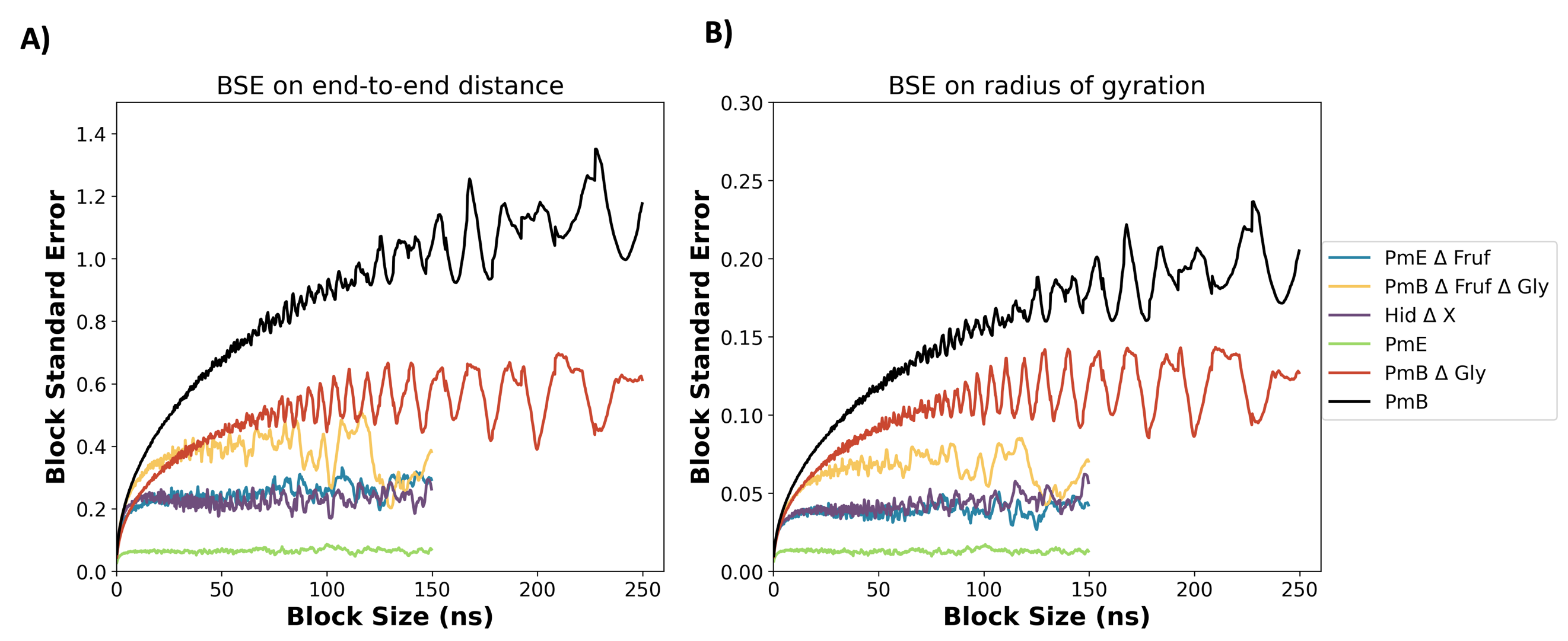


**Figure S 7:** Block standard averaging analysis for 6 RU modeled *P. multocida* and *H. influenzae* CPS molecules. (A) block standard error (BSE) versus block size (ns) calculated on end-to-end distance. (B) BSE versus block size calculated on radius of gyration. For all molecules, the BSE visually reaches a plateau.

| 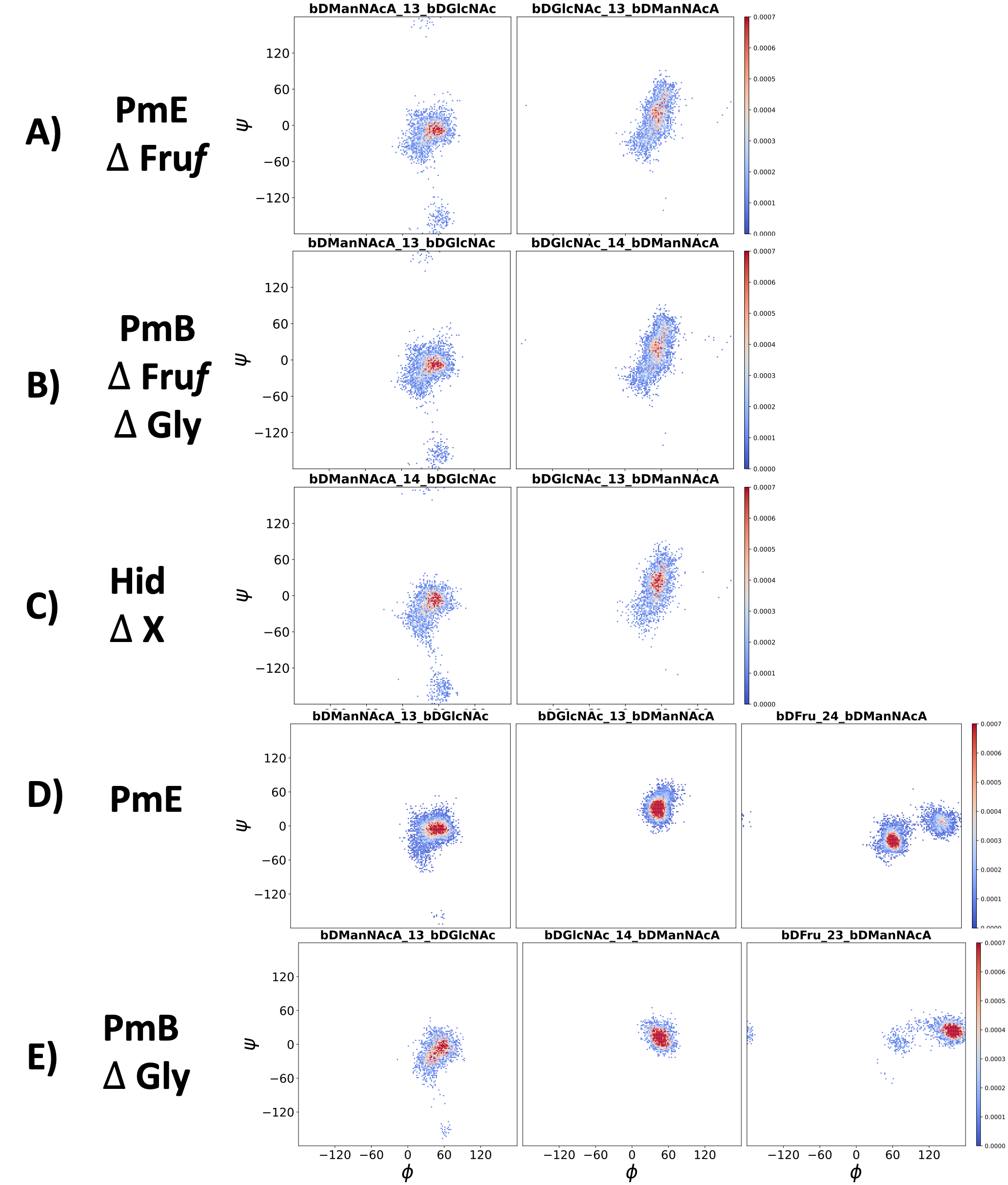  **Figure S 8:** Heatmap plots for the 3 RU molecules showing phi ($\phi$) vs psi ($\psi$) dihedral angles for ManNAcA🡪GlcNAc and GlcNAc🡪ManNAcA glycosidic linkages of: (A) PmE $\Delta$Fruf, (B) PmB $\Delta$ Fruf $\Delta$ Gly, and (C) Hid $\Delta$X as well as for the ManNAcA🡪GlcNAc and GlcNAc🡪ManNAcA and Fruf🡪ManNAcA linkages of (D) PmE, and (E) PmB $\Delta$Gly. X represents a variable amino acid moiety: L-alanine, L-serine, or L-threonine. The linkages were defined as we have done previously for these types of linkages (Richardson et al. 2022; Richardson et al. 2021). Glycosidic linkages were defined as φ = H_1_-C_1_-O_1_-C’_x_ and ψ = C_1_-O_1_-C’_x_-H’_x_. |
| --- |

|  |
| --- |

**References**

Haltiwanger RS. 2016. Symbol nomenclature for glycans (SNFG). *Glycobiology* 26(3):217.

Neelamegham S, Aoki-Kinoshita K, Bolton E, Frank M, Lisacek F, Lütteke T, O’Boyle N, Packer NH, Stanley P, Toukach P. 2019. Updates to the symbol nomenclature for glycans guidelines. *Glycobiology* 29(9):620-4.

Richardson NI, Kuttel MM, Ravenscroft N. 2022. Modeling of pneumococcal serogroup 10 capsular polysaccharide molecular conformations provides insight into epitopes and observed cross-reactivity. *Front Mol Biosci* 9:961532.

Richardson NI, Ravenscroft N, Arato V, Oldrini D, Micoli F, Kuttel MM. 2021. Conformational and immunogenicity studies of the shigella flexneri serogroup 6 O-antigen: The effect of O-acetylation. *Vaccines* 9(5):432.

Varki A, Cummings RD, Aebi M, Packer NH, Seeberger PH, Esko JD, Stanley P, Hart G, Darvill A, Kinoshita T. 2015. Symbol nomenclature for graphical representations of glycans. *Glycobiology* 25(12):1323-4.
